# Inflammatory cytokines promote growth of intestinal smooth muscle cells by induced expression of PDGF-Rβ

**DOI:** 10.1111/jcmm.12193

**Published:** 2014-01-13

**Authors:** Dileep G Nair, Kurtis G Miller, Sandra R Lourenssen, Michael G Blennerhassett

**Affiliations:** Gastrointestinal Diseases Research Unit, Department of Medicine, Queen's UniversityKingston, Ontario, Canada

**Keywords:** TNBS, smooth muscle, mitosis, PDGF, phenotype, stricture, colitis

## Abstract

Thickening of the inflamed intestinal wall involves growth of smooth muscle cells (SMC), which contributes to stricture formation. Earlier, the growth factor platelet-derived growth factor (PDGF)-BB was identified as a key mitogen for SMC from the rat colon (CSMC), and CSMC growth in colitis was associated with both appearance of its receptor, PDGF-Rβ and modulation of phenotype. Here, we examined the role of inflammatory cytokines in inducing and modulating the growth response to PDGF-BB. CSMC were enzymatically isolated from Sprague–Dawley rats, and the effect of tumour necrosis factor (TNF)-α, interleukin (IL)-1β, transforming growth factor (TGF), IL-17A and IL-2 on CSMC growth and responsiveness to PDGF-BB were assessed using proliferation assays, PCR and western blotting. Conditioned medium (CM) was obtained at 48 hrs of trinitrobenzene sulphonic acid-induced colitis. Neither CM alone nor cytokines caused proliferation of early-passage CSMC. However, CM from inflamed, but not control colon significantly promoted the effect of PDGF-BB. IL-1β, TNF-α and IL-17A, but not other cytokines, increased the effect of PDGF-BB because of up-regulation of mRNA and protein for PDGF-Rβ without change in receptor phosphorylation. PDGF-BB was identified in adult rat serum (RS) and RS-induced CSMC proliferation was inhibited by imatinib, suggesting that blood-derived PDGF-BB is a local mitogen *in vivo*. In freshly isolated CSMC, CM from the inflamed colon as well as IL-1β and TNF-α induced the early expression of PDGF-Rβ, while imatinib blocked subsequent RS-induced cell proliferation. Thus, pro-inflammatory cytokines both initiate and maintain a growth response in CSMC *via* PDGF-Rβ and serum-derived PDGF-BB, and control of PDGF-Rβ expression may be beneficial in chronic intestinal inflammation.

## Introduction

Inflammation of the intestine typically has rapid and dramatic consequences to organ function, including the appearance of pain, altered motility and perturbation of epithelial transport [1–2]. These can either be transient with full recovery or if more severe, can lead to lasting symptoms of altered motility that persist after inflammation is resolved [4]. The challenge to intestinal epithelium is an obvious effect, principally affecting the balance between secretion and absorption and thus leading to diarrhoea [5]. However, the underlying regions of the intestinal wall are also affected, and altered function of the smooth muscle layers and their associated neuronal plexuses is largely responsible for altered motility. In addition, animal models of intestinal inflammation as well as human disease reveal that incomplete reversal of these changes can leave lasting consequences to intestinal function [6].

Predictably, the severity of inflammation influences the extent of impact of the underlying events on the neuromuscular layers, which is seen in the contrast between the outcome of a superficial challenge that is fully reversed and the lingering outcome of severe enteritis [6]. These include the number, nature and location of activated immune cells and the diverse inflammatory factors that appear, which can broadly define the resulting inflammatory process as Th-1, Th-2 or Th-17 predominant [7, 8]. Although mild mucosal insults may be associated with primarily functional changes in epithelial cells, the relatively severe transmural inflammation of trinitrobenzene sulphonic acid (TNBS)-induced colitis (the principal animal model of Crohn's disease [9]) strongly affects the intestinal motor apparatus and can cause permanent damage such as the loss of myenteric and submucosal neurons [10]. While surviving myenteric neurons show axonal outgrowth that can restore innervation to the adjacent smooth muscle cells (SMC), there are also striking changes to that cell population: hyperplasia of intestinal smooth muscle cells (ISMC) leads to a nearly threefold increase in cell number that is not reversed [11]. This increase in potential force production is a challenge to intestinal motility, achieved by the regulation of excitatory and inhibitory neural input to smooth muscle. Furthermore, it is possible that progressive increase in SMC number is perpetuated in chronic inflammation and can participate in stricture formation [12, 13].

Smooth muscle proliferation also affects the nature and amount of extracellular matrix (ECM) and inflammatory change to these mesenchymal cells can lead to fibrosis. Elsewhere, fibrosis leads to the chronic loss of organ function [14], but the role of inflammatory change to the mesenchymal cells in intestinal fibrosis is still poorly understood. As inflammatory cytokines may both cause smooth muscle proliferation and influence fibrosis [15–16], better knowledge of their individual actions may explain how the changing profiles of these cytokines affect the outcome of intestinal inflammation.

The normally quiescent SMC can re-enter the cell cycle by modulation of phenotype and following cell division, redifferentiate fully. However, inflammation of either the vascular or pulmonary systems can lead to inappropriate growth that compromises organ function, through increased smooth muscle number as well as an altered contractile nature. Protracted smooth muscle proliferation has long been known to promote the loss of differentiated smooth muscle characteristics [18], along with a key role of inflammatory cells in promoting this [19]. However, the molecular basis of the events that cause pathological smooth muscle growth is not clearly understood, although numerous studies have shown an association among pro-inflammatory factors such as the cytokines IL-1β and TNF-α as well as mesenchymal growth factors such as insulin-like growth factor (IGF), FGF and platelet-derived growth factor (PDGF) [20–21].

The relevance of PDGF to the inflamed milieu, where platelets are activated [23], led us to earlier studies of inflammation-induced growth of the ISMC. A tissue culture model showed that the appearance of PDGF-Rβ was associated with proliferation of ISMC in the circular smooth muscle layer of the colon (CSMC), and that CSMC from the inflamed colon became responsive to PDGF [24]. Further work showed that PDGF-driven proliferation of CSMC *in vitro* led to the reduced expression of markers of the differentiated smooth muscle phenotype, which was also apparent among proliferating CSMC in acute colitis *in vivo* [25].

Therefore, we asked whether early events that are common to different forms of inflammation, such as the appearance of the pro-inflammatory cytokines, might influence smooth muscle growth directly or indirectly *via* promoting responsiveness to inflammation-derived factors. We evaluated this in culture models of either early-passage proliferating CSMC or freshly isolated CSMC. Here, we provide evidence that pro-inflammatory cytokines can drive the expression of the PDGF-Rβ among CSMC, and thus promote CSMC proliferation.

## Materials and methods

### Animals

Adult Sprague–Dawley rats (250–350 g) were obtained from Charles River (Quebec, Canada) and housed in pairs in microfilter-isolated cages for at least 5 days prior to use with free access to food and water. Colitis was induced by instillation of 500 μl of 200 mM TNBS (Fluka, Oakville, ON, Canada) dissolved in 50% ethanol into the colon 8 cm proximal to the anus, under light anaesthesia by inhalation of isoflurane as previously described [26]. Animals were killed by cervical dislocation under isoflurane anaesthesia as controls (untreated) or inflamed at 2 days after the initiation of colitis. All procedures received prior approval by the University Animal Care Committee of Queen's University.

### Tissue culture

Circular smooth muscle cells were obtained as previously described with minor modifications [24]. Briefly, small strips of circular smooth muscle tissue were removed from the mid-descending colon using fine dissection in HEPES-buffered Hanks' saline (pH 7.3), after removal of the mucosa and submucosa. These were enzymatically dissociated as described with muscle strips placed in a HEPES-buffered (HPSS) digestion solution containing papain (0.5 mg/ml; Sigma-Aldrich, Oakville, ON, Canada), BSA (1 mg/ml), *dl*-dithiothreitol (1 μM; Sigma-Aldrich) and collagenase type-F (0.5 mg/ml; Sigma-Aldrich) and incubated at 4°C for 2 hrs, and then room temperature for 1 hr and finally 37°C for 1 hr. The solution was replaced with DMEM containing 5% foetal calf serum (FCS; Invitrogen, Carlsbad, CA, USA), and cells were gently dissociated to produce a suspension of viable individual CSMC. For some experiments, the CSMC were dissociated in serum-free DMEM, with serum added subsequently as appropriate. Cells were then seeded in 24-well plates on either plastic wells (for immunoblotting and qPCR) or on rat tail collagen-coated glass coverslips (for immunocytochemistry). Phase contrast or Nomarski microscopy (Olympus IMT-2, IX-70; Olympus Canada, Richmond Hill, ON, Canada) was used to examine cell morphology of CSMC at various times following isolation. Cell viability was evaluated using trypan blue staining. Image acquisition and analysis were carried out using Image ProPlus 6.0 (Media Cybernetics, Rockville, MD, USA). Cytokines were obtained from PeproTech (Dollard des Ormeaux, QC, Canada) and PDGF-BB was obtained from Sigma-Aldrich.

To obtain conditioned medium (CM), equal amounts of muscularis externa were dissected from the colons of day 2 TNBS or control animals and incubated with serum-free DMEM for 24 hrs at 37°C [27]. The effects of the control or D2-CM on expression of PDGF-Rβ and proliferation were assessed by incubating passage 2 (P2) ISMC with CM for 24 or 48 hrs respectively.

### RT-PCR

Total RNA was isolated from freshly isolated ISMC or cells that were cultured for 1–7 days in DMEM with 5% FCS using an RNeasy kit (Qiagen, Toronto, ON, Canada) according to the manufacturer's protocol. Approximately 1 μg of total RNA after DNase I treatment was converted into cDNA using the iScript RT kit (Bio-Rad, Mississauga, ON, Canada). The expression of individual genes was analysed using GAPDH as the internal control. Primer sequences were PDGF-Rβ forward, 5′-CAACATTTCGAGCACCTTTGT-3′, and reverse, 5′-AGGGCACTCCGAAGAGGTAA-3′, yielding an amplicon of 677 bp; GAPDH forward, 5′-TGACAACTTTGGCATCGTGG-3′, and reverse, 5′-TACTCCTTGGAGGCCATGT-3′, producing a 513 bp amplicon; Cyclin D1 forward, 5′-TGGAGCCCCTGAAGAAGAG-3′, and reverse, 5′-AAGTGCGTTGTGCGGTAGC-3′, producing a 423 bp amplicon. The PCR conditions were 94°C for 4 min., then 94°C for 30 sec., 57°C for 30 sec. and 72°C for 45 sec. (31 cycles), and 72°C for 10 min. as final extension. The PCR products were resolved on a 1% agarose gel and imaged using Image Pro Plus 6.0 software (Media Cybernetics).

### Quantitative RT-PCR

cDNA (150–200 ng) obtained from ISMC were taken for real-time PCR using iTaq Fast SYBR Green supermix with ROX (Bio-Rad) in an Applied Biosystems 7500 Real-Time PCR instrument (Applied Biosystems, Burlington, ON, Canada). Primer sequences used were PDGF-Rβ forward, 5′-AATGACCACGGCGATGAGA-3′ and reverse, 5′-TCTTCCAGTGTTTCCAGCAGC-3′; GAPDH forward 5′-TGCACCACCAACTGCTTAG-3′ and reverse 5′-GATGCAGGGATGATGTTC-3′. Primers for cyclin D1 were forward, 5′-GCGTACCCTGACACCAATCT-3′; reverse, 5′-GGCTCCAGAGACAAGAACG-3′). Dissociation curve analysis following each amplification reaction confirmed the specificity of amplification. Expression of RNA was determined by the comparative threshold (Ct) analysis settings of the 7500 System Sequence Detection Software, version 1.3, using the ΔΔCt comparative method of relative quantification. Results were expressed as fold change relative to the freshly isolated cells.

For PrimeTime probe-based qPCR, cDNA (150–200 ng) was amplified by real-time PCR with 1× iTaq Universal Probes Supermix (Bio-Rad) using 500 nM of both forward and reverse primers and 250 nM of probe. Sequences for the amplification of PDGF-Rβ were Primer 1: 5′-CAGACTCAATGACCTTCCATCG-3′, Primer 2: 5′-GTACCACATCCTTGCCTTT-3′, probe: 5′-/56-FAM/TGGCAGAGG/ZEN/AAACCACGCTATGAG/3lABkFQ/-3′; cyclin D1 Primer 1: 5′ACCTCCTCTTCGCACTTCT-3′, Primer 2: 5′-GCCCTCCGTTTCTTACTTCA-3′, probe: 5′-/56-FAM/TCCTCGCAG/ZEN/ACCTCTAGCATCCA/3lABkFQ/-3′; and GAPDH Primer 1: 5′-CCAGTAGACTCCACGACATAC-3′, Primer 2: 5′-AACCCATCACCATCTTCCAG-3′, probe: 5′-/56-FAM/CAGCACCAG/ZEN/CATCACCCCATTTG/3lABkFQ/-3′. Primers were synthesized by Integrated DNA Technologies (Coralville, IA, USA). Expression of RNA was determined as above.

### Immunocytochemistry

Immunocytochemistry of CSMC in culture was carried out as previously described [24, 25]. Cells were fixed in 100% methanol to detect PCNA, washed and incubated overnight at 4°C with primary antibodies for PCNA (1/5000; Cell Signaling Technology PC10, New England Biolabs, Whitby, ON, Canada). They were then washed and incubated in appropriate ALEXA-linked secondary antibodies (1/1000 for 1 hr), as well as 1 μg/ml Hoechst 33342 for 30 sec. to label nuclei. The stained cells were visualized with fluorescence microscopy (Olympus BX51) and images were acquired using Image Pro Plus.

### Immunoblotting

To examine the expression and phosphorylation of PDGF-Rβ, immunoblotting was performed on confluent primary CSM according to cell number as reported earlier [25]. For this, an equal number of cells were first lysed in the presence of protease inhibitors and 1 μM vanadate. The protein samples were resolved by SDS-PAGE and transferred to polyvinyldifluoride membrane using a wet transfer apparatus (Bio-Rad). After transfer, blots were incubated for 1 hr at room temperature with blocking solution (5% non-fat milk in Tris-buffered saline containing 0.2% Tween-20). The membrane was then incubated with primary antibodies to PDGF-Rβ or phosphorylated-PDGF-Rβ (tyr751) overnight at 4°C (3161 and 3169 from Cell Signaling, 1/1000). After a brief wash, the blots were incubated in goat anti-rabbit horseradish peroxidase for 1 hr at room temperature. Bands were visualized using a chemiluminescent substrate (Super Signal, Pierce, Rockford, IL, USA) and the band intensity was determined using Image Pro Plus.

### Cell proliferation assay

Freshly isolated CSMC or early-passage CSMC were plated into 96-well microplates at 1 × 10^5^ cells/ml. Cell lines were serum-deprived for 24–48 hrs before stimulation by the addition of cytokines, PDGF-BB or serum. Cell proliferation was assessed after 2 days using the WST-8 assay (Cayman, Ann Arbor, MI, USA) according to manufacturer's instructions. Briefly, cells were incubated with WST-8 (2-methoxy-4-nitrophenyl)-3-(4-nitrophenyl)-5-(2,4-disulfophenyl)-2H-tetrazolium, monosodium salt) for 1 hr and the intensity of a colorimetric dye produced from WST-8 by viable cells was measured at 450 nm (EL-800; BioTek Instruments Inc., Fisher Scientific, Nepean, ON, Canada). Initial work validated the assay by comparison with cell number obtained by direct cell counting *via* haemocytometer.

### Statistics

Values are expressed as average ± SE of (*n*) experiments, where n is the number of animals. Statistical significance is assumed for *P* < 0.05 using one-way anova with post hoc analysis according to Tukey as appropriate.

## Results

### Promotion of growth of cultured CSMC

**Figure 1:**
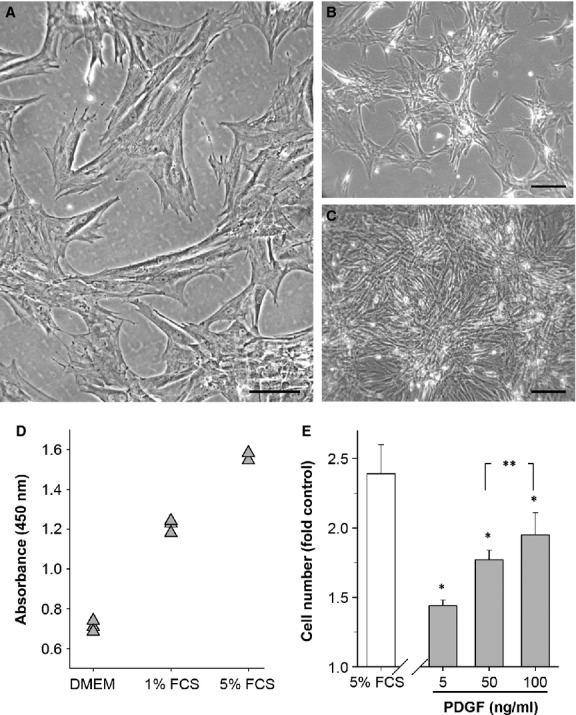
A culture model of proliferating adult colonic circular smooth muscle cells (CSMC) shows the mitogenic role of platelet-derived growth factor (PDGF)-BB. (A–C) Phase contrast images showing typical appearance of CSMC at passage 2 *in vitro* after plating (A), during growth (B) and at confluence (C). Scale bars: A, 100 μm; B and C, 200 μm. (D) Typical experimental outcome of CSMC growth after 48 hrs in either medium alone (DMEM) or DMEM + FCS using the Wst-8 assay. OD450 nm is proportional to cell number, shown here for triplicate wells per condition. (E) Average growth response of CSMC relative to serum-free control in either 5% FCS or PDGF-BB. All values significantly greater than control (*P* < 0.05). Cell number is expressed as the ratio of WST-8 reaction product in treated *versus* control wells, for FCS (*n* = 8) or PDGF-BB (*n* = 4), with triplicate wells per experiment. *P < 0.05 *versus* control; **P > 0.05.

After isolation from the circular muscle layer of the adult rat colon, CSMC attached readily to the culture surface, modulated their appearance and proliferated, as reported earlier [24, 25]. Upon subculturing, these CSMC were typically pleomorphic at low initial density, but quickly assumed a collinear, branching appearance as growth progressed (the ‘hill and valley’; Fig. 1A–C). The Wst-8 assay of cell number showed that FCS caused a large, reproducible increase in signal by 48 hrs compared with unstimulated (DMEM alone) time-matched controls (*e.g*. Fig. 1D), while those controls showed no change in cell number over the duration of the experiment (data not shown). In parallel studies, addition of PDGF to DMEM caused a concentration-dependent increase in cell number that was reproducible among cell lines isolated from different animals (Fig. 1E). This demonstrated that mitogens were capable of promoting cell proliferation in serum-free medium, at least over this time period, and established the basis for subsequent studies.

**Figure 2:**
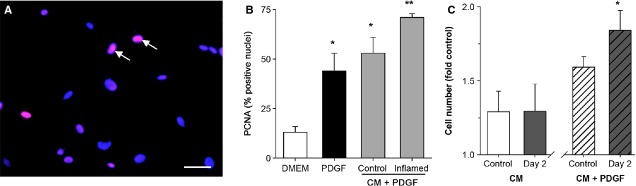
Conditioned medium (CM) from the inflamed intestine does not promote growth of circular smooth muscle cells (CSMC) directly, but is mitogenic *via* platelet-derived growth factor (PDGF)-BB. (A) Representative image of CSMC culture stained for expression of the proliferation marker PCNA and counter-stained for nuclei using H333252, showing positive nuclei (magenta; arrows) among predominantly negative nuclei (blue). Scale bar, 40 μm. (B) Quantification of increased expression of PCNA in CSMC exposed to PDGF-BB (10 ng/ml) or PDGF-BB combined with CM from control or inflamed colon. CM from inflamed but not control colon increased PCNA expression above the effect of PDGF-BB alone (*P < 0.05 *versus* control medium (DMEM); **P < 0.05 *versus* control CM + PDGF-BB). (C) CSMC proliferation assay showing that CM from control rat colon or from colon at Day 2 of TNBS-induced colitis did not increase CSMC proliferation (left; *P* > 0.05 *versus* untreated control). However, CM from the inflamed colon significantly increased growth when combined with PDGF-BB (right; *P < 0.05).

To test whether the milieu of the inflamed intestine could stimulate CSMC growth, CM was obtained from the smooth muscle/myenteric plexus of either the control or inflamed colon (Day2 post-TNBS). This time-point reflects a phase of acute inflammation in this model, when infiltrating immune cells and pro-inflammatory cytokines are prominent, and was selected to assess the presence of factors that might initiate CSMC proliferation, which is apparent at this time (see Fig. 5) [11]. First, immunocytochemistry was used to test for the ability of CM to stimulate the appearance of the mitotic marker PCNA by 24 hrs after application, but no significant increase in expression was detected, although present with the positive control of PDGF (Fig. 2). However, addition of PDGF to inflamed CM caused a significantly greater effect than either inflamed CM or PDGF alone, while control CM had no significant effect (Fig. 2B). When this was further tested for the ability to support CSMC proliferation by 48 hrs, inflamed CM significantly increased cell number when PDGF was added, but not otherwise (Fig. 2C). This suggested that inflamed CM was not itself a significant mitogenic influence, but could promote the action of a mitogen.

**Figure 3:**
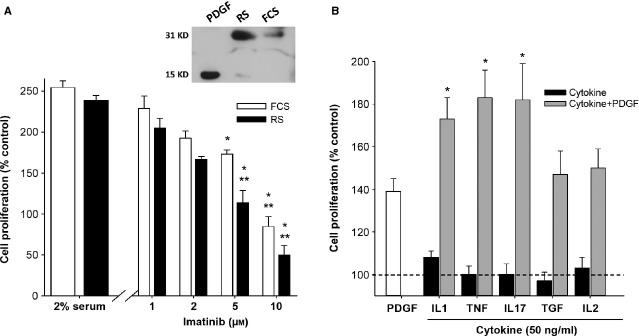
Homologous serum and inflammatory cytokines promote circular smooth muscle cells (CSMC) growth *in vitro via* platelet-derived growth factor (PDGF). (A) Proliferation of CSMC in cohort cultures exposed to either 2% FCS or 2% adult rat serum (RS) for 48 hrs. The effect of RS was reduced to control levels by the PDGF-Rβ inhibitor imatinib (*P < 0.05 *versus* RS; **P > 0.05 *versus* serum-free control; *n* = 4). Inset, representative image of immunoblot showing the presence of PDGF-BB in both FCS and RS as a 31 KD dimer, with detectable monomer (15 KD) in RS. Left lane, recombinant PDGF-BB. (B) Pro-inflammatory cytokines did not affect growth of CSMC directly, but strongly promoted the effect of PDGF-BB. Replicate cultures of CSMC were synchronized and exposed to PDGF-BB (10 ng/ml), cytokine (50 ng/ml) or cytokine + PDGF-BB, and cell proliferation was evaluated 2 days later. In triplicate cultures from three independent experiments, there was no direct effect of any cytokine (black bars), while the pro-inflammatory interleukin (IL)-1β, tumour necrosis factor (TNF)-α and IL17A significantly increased the response of cells to PDGF-BB (grey bars; *P < 0.05 compared with PDGF-BB alone).

In studying this further, we reasoned that sites of inflammation are characterized by increased endothelial leakiness and immune cell immigration as well as platelet activation that would liberate PDGF. The serum fraction of blood that is allowed to clot *ex vivo* represents the contribution of blood components, but without the inflammatory factors derived from a local host immune response. Therefore, serum from adult control rats (RS) was tested for the ability to cause CSMC growth. In this growth assay, RS was highly effective as a mitogen and comparable to traditionally used FCS (Fig. 3A). To identify the role of PDGF in serum-stimulated growth, the PDGF-Rβ inhibitor imatinib (a multiple tyrosine kinase inhibitor with selectivity for the PDGF-Rβ isoform [28–29]) was added 20 min. prior to stimulation and the outcome of cell growth assessed 2 days later. Imatinib caused the concentration-dependent inhibition of both RS-and FCS-dependent growth (Fig. 3A), indicating a prominent role for PDGF-Rβ. As further confirmation of the role of PDGF, western blotting consistently showed the presence of dimeric PDGF-BB in FCS and RS (inset, Fig 3A), where specificity was established using the positive control of recombinant PDGF-BB, antibody omission and inappropriate secondary antibodies, with both monomer and dimeric forms present under mild reducing conditions. As earlier studies showed that adult rat CSMC were not significantly responsive to growth factors such as IGF, epidermal growth factor or PDGF-AA [24], this indicated that PDGF-BB is an important mitogen for CSMC in both RS and FCS, and supported further investigation into the modulation of PDGF-Rβ in the inflamed intestine.

Separately from the serum-derived effects, CM-derived *ex vivo* from inflamed, but not control, colonic tissue did not have a direct effect on CSMC proliferation. This suggested that the immune cells present in the inflamed tissue were involved and so a range of cytokines were examined for their effects on CSMC. Surprisingly, no cytokine caused a significant increase in CSMC proliferation when tested alone, at levels up to 100 ng/ml (Fig. 3B, black bars). However, the combination of PDGF with each of the pro-inflammatory cytokines IL-1β, TNF-α or IL-17A caused a striking increase in CSMC growth over PDGF alone (Fig. 3B, grey bars). In contrast, the cytokines transforming growth factor (TGF) and IL-2 remained ineffective, showing that this action was limited to these prototypic pro-inflammatory factors.

**Figure 4:**
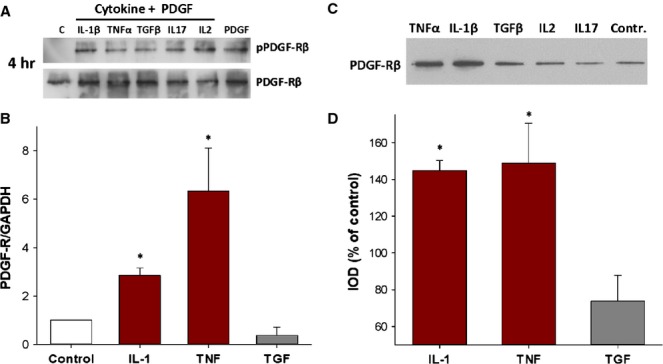
Pro-inflammatory cytokines cause increased expression of platelet-derived growth factor (PDGF)-Rβ without promoting receptor activation. (A) Cytokine treatment did not affect the phosphorylation of PDGF-Rβ by PDGF-BB. Representative images of phosphorylation assay showing similar outcome after treatment with PDGF-BB of control or cytokine-treated circular smooth muscle cells (CSMC). Cells were treated with cytokine (50 ng/ml) for 4 hrs before addition of PDGF-BB (50 ng/ml) for 15 min. C, untreated control. (B) Outcome of qPCR showing significant increase in mRNA for PDGF-Rβ in CSMC at 17 hrs after exposure to interleukin (IL)-1β or tumour necrosis factor (TNF)-α *versus* control CSMC (*P < 0.05 *versus* untreated control). (C) Representative image of immunoblot showing increased expression of PDGF-Rβ in CSMC treated with IL-1β or TNF-α (50 ng/ml) for 24 hrs. Loading was normalized through a constant cell number (5000 CSMC) per lane. (D) Quantification of western blotting of CSMC showing increased expression of PDGF-Rβ with IL-1β or TNF-α treatment (*P < 0.05; *n* = 3), while no increase occurred with transforming growth factor (TGF)-β (*P* > 0.05).

Cytokines could potentiate the outcome of addition of PDGF by affecting the efficiency of receptor activation and/or by alteration in receptor amount. To distinguish these, CSMC cultures were pre-treated with cytokines for either 4 or 24 hrs, stimulated with PDGF for 15 min. and examined by western blotting for receptor phosphorylation. There was no evidence of altered pPDGF-Rβ amounts compared with total PDGF-Rβ among these outcome (*e.g*. Fig. 4A). However, by 17 hrs after cytokine application, qPCR showed significantly increased mRNA levels for PDGF-Rβ in CSMC treated with IL-1β or TNF-α, but not TGF (Fig. 4B). This evidence for increased receptor expression was verified by western blotting, which showed that both IL-1β and TNF-α caused marked up-regulation of PDGF-Rβ protein, while TGF, IL-2 and IL-17A were without effect (representative image, Fig. 4C, and averaged outcome, Fig. 4D). These experiments were normalized by loading a constant cell number, which directly relates the signal to cellular contents and avoids the possibility of alterations because of changes in CSMC size or activity, as shown previously [24, 25].

This supports the conclusion that the inflamed environment both contains PDGF and promotes its signalling *via* up-regulation of PDGF-Rβ. This suggests that a PDGF-mediated process is responsible for the growth of CSMC during inflammation, and thus the outcome of increased CSMC number. However, earlier studies showed that control rat CSMC do not express PDGF-Rβ, and that expression arises early in the time course of inflammation in the TNBS model of colitis [24]. This raised the question of whether inflammatory factors alone might also be able to accelerate or even cause the appearance of PDGF-Rβ. To pursue this, we examined the role of the PDGF pathway at early times following the isolation of control CSMC, as they adapt to culture and initiate expression of PDGF-Rβ.

### Accelerated onset of growth in freshly isolated CSMC

**Figure 5:**
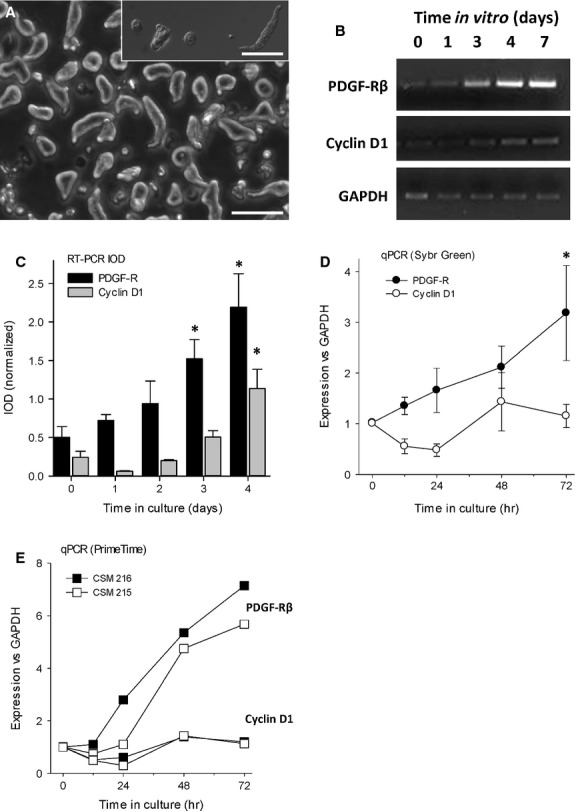
The appearance of mRNA for platelet-derived growth factor (PDGF)-Rβ in freshly isolated adult circular smooth muscle cells (CSMC) precedes entry into cell cycle. (A) Appearance of CSMC at 6 hrs after isolation, showing transition from a long, bipolar appearance to a rounded, surface-attached appearance. Phase contrast main image (scale bar, 100 μm) and inset, Nomarski optics (scale bar, 80 μm). (B) Representative image of RT-PCR experiment showing appearance of mRNA for PDGF-Rβ occurs before that of cyclin-D1. (C) Quantification of RT-PCR showing time course of onset of expression of PDGF-Rβ in freshly isolated CSMC from control rats, with outcome normalized to GAPDH. CSMC initially lack significant expression of PDGF-Rβ, which first appeared by Day 3 and increased thereafter (*P < 0.05 *versus* control). (D) Quantitative PCR showing that the appearance of mRNA for PDGF-Rβ precedes mRNA for cyclin D1, a marker of proliferation. Data are the means of three experiments (*i.e*., cells from three separate animals) with two replicate wells per time-point (*P < 0.05 *versus* control). (E) Outcome of two independent experiments showing time course of up-regulation of mRNA for PDGF-Rβ, which consistently preceded that of cyclin D1. Data are the average of two wells per condition per animal, assessed by qPCR using PrimeTime probes.

If PDGF-Rβ is both necessary and sufficient for CSMC proliferation, it should be expressed before the onset of cycle progression, and inhibition should prevent cell proliferation. Using freshly isolated control CSMC, we first determined the time of onset of expression of mRNA for PDGF-Rβ in comparison with the appearance of mRNA for the critical cell-cycle progression marker cyclin D1 [31, 32]. Three methods were used, end-point PCR (RT-PCR; optimized for cycle number), qPCR using Sybr Green detection of amplified DNA and by PrimeTime probe-based qPCR. In all cases, the outcome showed a significant increase in mRNA for PDGF-Rβ at approximately 2–3 days *in vitro* and always *before* that of cyclin D1 (Fig. 5). This showed that expression of this receptor precedes the onset of cell division, and is not a consequence of it.

**Figure 6:**
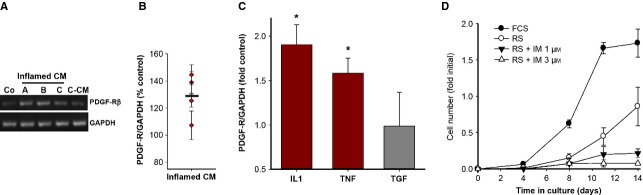
Inflammatory factors cause growth of freshly isolated control adult circular smooth muscle cells (CSMC) *via* accelerated expression of platelet-derived growth factor (PDGF)-Rβ. (A) Typical outcome of RT-PCR of CSMC at 6 days after isolation from the control colon, showing accelerated appearance of mRNA for PDGF-Rβ in CSMC exposed to conditioned medium (CM) from the inflamed intestine (inflamed CM) *versus* control CM (C-CM). (A–C) Separate experiments testing inflamed CM from each of three animals; Co, untreated control CSMC. (B) Quantification of appearance of mRNA for PDGF-Rβ among CSMC from each of four animals at 6 days *in vitro*, exposed to CM from Day 2 of TNBS-colitis. Each data point shows average response ±SE Horizontal bar, mean response (*P* < 0.05 compared to control). (C) qPCR outcome showing that serum-free DMEM containing the pro-inflammatory cytokines interleukin (IL)-1β or tumour necrosis factor (TNF)-α but not transforming growth factor (TGF)-β promoted appearance of mRNA for PDGF-Rβ in freshly isolated CSMC by Day 3 *in vitro* (*P < 0.05 *versus* DMEM alone; *n* = 3). (D) Adult rat serum causes proliferation of freshly isolated adult CSMC *via* PDGF-Rβ. Cohort cultures were grown in either 2.5% FCS, 2.5% RS or RS with the PDGF-Rβ inhibitor imatinib. Cell proliferation stimulated by RS (≥Day 8; *P* < 0.05) was completely blocked by imatinib without toxicity.

We next tested whether CM from the inflammatory milieu affected expression of PDGF-Rβ. Freshly isolated CSMC that were incubated with inflamed CM showed accelerated expression of mRNA for PDGF-Rβ compared with either control CM or untreated control cells (Fig. 6A and B). Critically, qPCR at 3 days *in vitro* showed that IL-1β and TNF-α, but not TGF, significantly increased message for PDGF-Rβ in freshly isolated CSMC, maintained in serum-free DMEM (Fig. 6C).

Finally, the necessity of the PDGF receptor for the onset of CSMC growth was shown through inhibition of PDGF-Rβ-mediated signalling with imatinib (1 and 3 μM). Receptor inhibition did not cause cytotoxicity and the CSMC were observed to attach and adhere normally. Strikingly, this prevented the proliferation of CSMC in response to RS, which was otherwise prominent by day 14 *in vitro* (Fig. 6D).

These studies of the early events of the onset of proliferation among isolated CSMC indicate that the appearance and function of PDGF-Rβ is a necessary first step. The initiation of the process by pro-inflammatory cytokines, its acceleration by inflamed CM and the role of homologous adult serum support the conclusion that inflammation causes smooth muscle proliferation similarly *in vivo* by first inducing receptor expression and then supporting its activation.

## Discussion

The thickened intestinal wall is characteristic of inflammation, in human disease and in animal models of intestinal inflammation [33–34]. As part of the debilitating consequences of chronic human inflammatory bowel disease, this can lead to obstruction and a recurrent need for intestinal resection. In Crohn's disease and in TNBS-induced colitis, the animal model that closely models Crohn's, inflammatory processes cause smooth muscle proliferation as the major cellular component of bowel wall thickening [11]. These changes to the smooth muscle layers are an important secondary feature of the inflammation and gaining selective control of this pathology is a desirable medical advance.

Here, we studied the effects of inflammation on growth of intestinal smooth muscle, using CSMC from the rat colon to examine the role of the smooth muscle growth factor PDGF and its receptor, PDGF-Rβ. In recent work, we showed that PDGF-Rβ is normally absent from control CSMC, but appears in colitis [24]. Furthermore, we showed both the presence of PDGF-Rβ on CSMC by immunocytochemistry and PDGF-BB–induced phosphorylation of its receptor [24]. In this study, we found that pro-inflammatory cytokines exerted a critical effect on CSMC through stimulating the expression of PDGF-Rβ in quiescent control cells and then further promoting the resulting growth response through up-regulating expression of the PDGF-Rβ. Thus, the pro-inflammatory cytokines increased the proliferation of CSMC in response to PDGF-BB, but did not have any effect when added alone. PDGF-BB was found to be the major mitogen in both rat and FCS, and thus platelet activation during inflammation *in vivo* would liberate a potent growth factor in proximity to responsive cells. Therefore, the inflammatory milieu can both initiate receptor expression and then drive its activation to cause CSMC proliferation.

Adult differentiated SMC remain able to switch from the contractile state to a synthetic or proliferative state, which is marked by proliferation, migration and altered matrix synthesis [36]. However, studies of SMC proliferation *in vitro*, particularly of vascular SMC but recently of intestinal SMC, have shown both a progressive loss of differentiated characteristics and a reduced ability to redifferentiate upon removal of the mitogenic stimuli [25, 37]. Therefore, inflammation that causes growth of the SMC can pose a considerable challenge to organ function, a significant factor in several human diseases. In the airway, pulmonary arterial hypertension is caused by medial hypertrophy as a result of proliferation of pulmonary arterial smooth muscle and is associated with inflammation [38]. Both atherosclerosis in humans and the outcome of experimental endothelial damage in animal models include local SMC migration, modulation of phenotype and proliferation that creates vessel wall thickening [39].

The effects of smooth muscle proliferation in the intestine are less well understood. As these cells create the peristaltic pressure waves that are responsible for appropriate propulsion of intestinal luminal contents, an increased number of cells with an altered contractile nature are both an acute challenge to normal intestinal motility and a cumulative risk for future obstruction. The challenge to both intrinsic and extrinsic innervation may also contribute to symptoms of post-infectious functional bowel disease (irritable bowel syndrome).

The association of inflammation and smooth muscle growth has led to study of the pro-inflammatory cytokines, particularly in the vascular system [39–40]. For example, IL-1β and other inflammatory cytokines are not typically mitogenic (although this may vary according to tissue [42–43]), but modulate the SMC phenotype towards the decreased expression of smooth muscle marker genes such as smooth muscle α-actin, myosin heavy chain, SM22α and calponin, along with increased expression of ECM, adhesion molecules and inflammatory cytokines such as IL-6 – an inflammatory phenotype [21, 45]. Notably, the growth factor PDGF contributes directly to the atherosclerotic process by promoting vascular smooth muscle proliferation and migration, but also PDGF acts *via* its receptor, PDGF-Rβ, to cause a well-recognized suppression of smooth muscle marker genes [46]. Thus, our finding that PDGF is the predominant growth factor in adult homologous serum for the intestinal SMC, which can alone drive proliferation and thus modulate the ISMC phenotype, establishes the key structural elements for a similar process in intestinal inflammation. This emphasizes the critical and specific role for appearance of the PDGF-Rβ on the general ISMC population, rather than a PDGF-responsive subtype like that found elsewhere [47].

In both TH-2 and TH-1 dominant models of experimental intestinal inflammation, smooth muscle growth causes lasting increase in cell number and size [34, 35, 48], but the cellular mechanisms and the consequences to intestinal function are much less clear. Comparison between freshly isolated and proliferating cells *in vitro* showed a rapid loss of phenotypic markers that became exaggerated with further cell division [25], similar to changes described in vascular SMC both during growth *in vitro* and in atherosclerosis *in vivo*. A similar change in phenotype was seen *in vivo*, when FACS was used to isolate proliferating CSMC from the inflamed intestine, proving that a parallel process occurs *in vivo*.

Improved treatments of IBD and in particular of fibrostenotic Crohn's disease have made great strides in patient care, but these ultimately remain symptomatic: in most cases, the natural history of CD continues to play out its cycle of remission and exacerbation. This raises the possibility that a novel cellular phenotype arises, which can over time promote the resurgence of disease either directly or more reasonably, indirectly by a lowered threshold and increased capacity for response to normally insignificant challenges. This idea may explain the recent finding that bowel wall thickness at time of surgery for CD was a predictor of repeated surgical intervention [49]. In support, recent work suggests that the increased ECM seen in CD may directly promote further fibrogenesis [50]. Of particular concern, the enlarged population of CSMC that arose in the TNBS model of colitis displayed an exaggerated growth response when cultured; even weeks after inflammation had resolved *in vivo* [25]. This points to the SMC cell as a potentially active participant in the outcome of chronic intestinal inflammation.

Are all affected cells equal, and do all respond? Studies of contractility at the single cell level suggest that cells from a given layer, *e.g*. circular or longitudinal, are equivalent (*e.g*. [26]). However, there remains the possibility for a cryptic, responsive subset of CSMC or the contribution of unsuspected cells with stem-like capacity. Our work has used exclusively enzymatically isolated single cells and not explant-derived cells. From this, we concluded that most if not all isolated cells can and do contribute to the primary cell cultures, both from following the original input isolated CSMC population by time-lapse photography, and by direct counting of responsive cell numbers. While the suggestion of a multi-potent vascular stem cell in the blood vessel wall was intriguing and potentially relevant [51], this was refuted by Nguyen *et al*. [52]. This idea of a small, stem-like cell population does not fit our direct observations, and we conclude that the CSMC retain the ability to respond to an inflammatory insult by reversible dedifferentiation and proliferation.

The circumstances in the intestine that dictate the onset of growth still remained unclear. The pro-inflammatory cytokines are key factors, as they render the CSMC responsive to PDGF and then promote that response. These cytokines are a hallmark of the onset of TNBS-induced colitis [53] and extrapolating from the presence of PDGF in serum, platelet activation in the inflamed intestinal wall would liberate PDGF into the CSMC microenvironment. The inflamed milieu (as represented by CM from Day 2 of colitis) thus comprises both instructive and permissive aspects for PDGF-Rβ–dependent CSMC growth. In the later phases of inflammation, a relative upswing of pro-fibrotic factors such as the cytokine TGF-β might then further modulate the expression profiles of a susceptible and now-increased population of SMC. In this way, the repetitive events of chronic relapsing inflammation might drive stricture formation.

Other factors are also important, and the interaction among these is not yet clear. For example, we showed that damage to innervation of the CSMC, which is a prominent aspect of colitis coincident with CSMC proliferation, promotes proliferation *via* removal of a normally inhibitory nitrergic input [54, 55]. The tonic input of nitric oxide, whether derived from the endothelium in the vasculature or *via* nitrergic enteric neurons, is thus a common regulatory event. Furthermore, SMC both secrete and respond to ECM factors and decreased expression of collagen I and III, with increased collagen VIII and ICAM-1 reflects the modulated SMC phenotype [39, 56]. The IGF and their binding proteins play significant roles, and experimental reduction in IGF-I is instrumental in reducing intestinal smooth muscle hyperplasia and excess collagen production in TNBS-induced colitis in mice [57].

At the subcellular level, the SMC must integrate multiple signals in the transition from quiescence to an inflammatory phenotype. Common co-ordinating factors such as NF-κB mobilization and transcription factors such as Kruppel-like factor 4, myocardin and ETS domain-containing protein play vital roles in SMC growth in other systems and are expected to have critical roles in ISMC as well. Their integration into a clear picture of the reasons for and consequences of ISMC growth remains a future goal. As this becomes resolved, the selective retention of beneficial outcome along with suppression of deleterious consequences can assist our ability to usefully interfere in the inflammatory response.
